# Epidemiology of *Candida albicans* and *non-C.albicans* of neonatal candidemia at a tertiary care hospital in western China

**DOI:** 10.1186/s12879-017-2423-8

**Published:** 2017-05-06

**Authors:** Jinjian Fu, Yanling Ding, Ba Wei, Lin Wang, Shaolin Xu, Peixu Qin, Liuhua Wei, Lijun Jiang

**Affiliations:** 1 Department of Laboratory, Liuzhou Maternity and Child Healthcare Hospital, Liuzhou, China; 2 Department of Neonatology, Liuzhou Maternity and Child Healthcare Hospital, Liuzhou, China; 3 Department of Science and Education, Liuzhou Maternity and Child Healthcare Hospital, Liuzhou, China; 4 Department of Laboratory, Liuzhou Worker’s Hospital, No 1 Liushi Rd, Liuzhou, 545005 China

**Keywords:** Infants, Candidemia, Non-C.albicans, Assisted reproductive technology

## Abstract

**Background:**

Although the majority of *Candida* infections occur in the developing world, candidemia epidemiology is poorly understood in these countries. The aim of this study was to investigate the epidemiology of *non-Candida albicans (non-C. albicans)* candidemia among neonates at Liuzhou Maternity and Child Healthcare Hospital in China.

**Methods:**

A retrospective review of all positive blood culture about *Candida* species in neonatal intensive care unit was conducted between January 2012 and November 2015. Information about demographics, risk factors and outcome of candidemia were collected. Univariate and multivariate logistic regression models were used to identify the risk factors associated with the development of *non-C.albicans* candidemia.

**Results:**

The prevalence of candidemia in infants was 1.4%. *Non-C.albicans* was responsible for 56.5% of neonatal candidemia. The predisposing factors for development of *non-C.albicans* candidemia among infants included mechanical ventilation [odds ratio (OR), 95% confidence interval (95%CI) = 3.13, 1.07–9.14; *P* = 0.037] and use of assisted reproductive technology (OR, 95%CI = 4.52, 1.39–14.77; *P* = 0.012). The overall mortality rate of candidemia was 8.7% and *non-C.albicans* attributed to 83.3% of all mortalities.

**Conclusions:**

*Non-C.albicans* species are the major cause of candidemia in local neonatal group. The study highlights the urgent needs to evaluate the possibility of development of *non-C.albicans* candidemia in neonates exposed to these risk factors and much emphasis must be laid on the early implementation of medical intervention to reduce the incidences of candidemia in neonates.

## Background

*Candida* species are the third most common pathogens that attributed to neonatal bloodstream infections and are associated with 20–34% mortality [1, 2]. Although *Candida albicans* has long been the most common cause of candidemia infections obtained from neonatal intensive care units (NICUs) [3, 4]. *Non-C.albicans* species such as *Candida glabrata (C.glabrata)*, *Candida tropicalis (C.tropicalis)*, *Candida parapsilosis* (*C.parapsilosis*) and *Candida krusei* (*C.krusei*) have emerged as the second or even the predominant pathogens that caused neonatal candidemia in some local hospitals [5].

Infants are more vulnerable to develop candidemia due to their critical underlying conditions, immature immune system, and invasive operations [6, 7]. The predisposing factors contributing to the current increase of neonatal candidemia included prematurity, very low birth weight, aggressive broad-spectrum antibiotics, prolonged hospitalization, invasive operations such as central venous catheters, intubation and total parenteral nutrition [8–12].

As the *Candida* species formed the biofilms on the surface of indwelling medical appliances, their high virulence, the documented horizontal nosocomial transmission, vertically from mother to neonate, and their developmental of resistance to azole antifungal agents [8, 9, 13], the neonatal candidemia has presented a challenge to pediatricians.

Although several studies had reported the epidemiology of *Candida* infections in China, they mainly focused on adults, invasive *Candida* infections or conducted among special groups [14–17]. The investigation about *Candida albicans* (*C.albicans*) compared to *non-C.albicans* candidemia in neonatal groups are limited in China [18]. It is important to know local information about candidemia infections in NICUs as the *Candida* infections at local levels could provide pediatricians the full information to implement early medical practice to diagnosis and treat these high-risk groups. This retrospective study aimed to investigate the epidemiology of candidemia in neonatal groups at a single center in western China.

## Methods

### Study design and data collection

This retrospective study was conducted between January 2012 and November 2015, in NICU of Liuzhou Maternity and Child Healthcare Hospital. The medical charts for 5075 admissions during the 4-year period were reviewed. Infants with positive blood culture results of *Candida* species were defined as candidemia. Collected demographics on previously identified risk included necrotizing enterocolitis, neurodevelopmental impairment, maternal underlying diseases (such as preeclampsia, gestational diabetes, pregnancy-induced hypertension, cholestasis, hyperthyroidism, hypothyroidism, etc.), respiratory distress, vaginal birth, fetal membrane rupture, neonatal congenital diseases (such as congenital heart disease, Glucose-6-Phosplate Dehydrogenase deficiency, thalassemia, etc.), abdominal surgery, mechanical ventilation, central venous catheter, intubation, pulmonary active substance use, steroids use, antacid use, total parenteral nutrition, hospitalization duration, 3rd cephalosporins use, carbapenems use, vancomycin use, multiple antibiotic (≥3 classes) use, antibiotic therapeutic duration (including the administration of antibiotics prior to the occurrence of candidemia and the prophylaxis antibiotic use during hospitalization), use of prophylaxis antifungal agent (fluconazole), and antifungal therapeutic duration. The collection data from medical chart was permitted by the Institutional Review Board.

### Microbiologic methods

Blood samples were cultured in the BacT/AlerT 3D system (Biomérieux). Chromo Agar medium was used for Candida species identification (Biomérieux) and API 20C AuX (Biomérieux) was used for confirmation.

### Statistical analysis

SPSS version 20.0 statistical software (SPSS Inc., Chicago, Il, USA) was used. Potential risk factors associated with increased candidemia risk were identified using univariate logistic regression analysis. Variables with 2-tailed *P* < 0.05 were included in the multivariate logistic regression model to evaluate the odds ratios (ORs) and 95% confidence intervals (CIs) to calculate the strength of any association. A *P*-value less than 0.05 was considered indicative of statistical significance.

## Results

### The distribution of Candida species and incidence of candidemia

There were 5075 admissions of neonates (aged <28 days) in the NICU, 69 infants had *Candida* bloodstream infections during the 4-year study period. The incidence of candidemia was 1.4%. *C.albicans* was the leading causative pathogen of candidemia and it was isolated in 43.5% of the patients. *Non-C.albicans* was responsible for 56.5% of neonatal candidemia. The distribution of *non-C.albicans* species was *C.glabrata* for 33.3%, *C.tropicalis* for 20.3%, *C.parapsilosis* for 1.4% and *C.kefyr* for 1.4%.

### Risk factors associated with candidemia due to non-C.Albicans

Infant demographics and risk factors for candidemia are shown in Table 1. There was no difference between *C.albicans* and *non-C.albicans* in terms of the gestational age (31.7 vs 31.5 weeks, *P* = 0.765), birth weight (1670.0 g vs 1417.6 g, *P* = 0.128), or male gender (16.7% vs 30.8%, *P* = 0.150).

**Table 1:** Clinical characteristics of neonates between *C.albicans* and non-*C.albicans*

Variable	*C.albicans* mean (95%CI) or n (%)	non-*C.albicans* mean (95%CI) or n (%)	*P* value	odds ratio (OR) (95%CI)
Demographics				
gestational age (wks)	31.7 (27.6, 35.8)	31.5 (27.7, 36.3)	0.765	
birth weight (g)	1670.0 (962.1,2377.9)	1417.6 (777.1,2058.1)	0.128	
male gender, n (%)	22 (73.3)	22 (56.4)	0.150	0.47 (0.17–1.32)
admission age	2.1 (2.0, 6.2)	1.1 (0.6, 1.6)	0.138	
Risk factors				
necrotizing enterocolitis	5 (16.7)	12 (30.8)	0.183	2.22 (0.69–7.21)
neurodevelopmental impairment	7 (23.3)	8 (20.5)	0.778	0.85 (0.27–2.68)
maternal underlying diseases	12 (40.0)	18 (46.2)	0.609	1.29 (0.50–3.36)
assisted reproductive technology	5 (16.7)	19 (48.7)	0.008	4.75 (1.51–14.96)
vaginal birth	19 (63.3)	23 (59.0)	0.713	1.20 (0.45–3.20)
fetal membrane rupture (h)	16.4 (28.4, 61.2)	26.6 (36.0, 89.2)	0.459	
congentital diseases	13 (43.3)	24 (61.5)	0.135	2.09 (0.80–5.51)
abdominal surgery	4 (13.3)	6 (15.4)	0.811	1.18 (0.30–4.63)
mechanical ventilation	14 (46.7)	29 (74.4)	0.021	3.31 (1.20–9.15)
central venous catheter	14 (46.7)	27 (69.2)	0.061	2.57 (0.96–6.91)
intubation	10 (33.3)	21 (53.8)	0.092	2.33 (0.87–6.26)
pulmonary active substance use	7 (23.3)	9 (23.1)	0.980	0.99 (0.32–3.04)
steroids use	6 (20.0)	9 (23.1)	0.759	1.20 (0.38–3.84)
antacid use	3 (10.3)	8 (21.1)	0.250	2.31 (0.56–9.63)
total parenteral nutrition	23 (76.7)	36 (92.3)	0.080	3.65 (0.86–15.57)
hospitalization duration (d)	43.8 (19.1, 78.5)	49.1 (26.6, 71.6)	0.367	
3^rd^cephalosporins use	16 (55.2)	23 (59.0)	0.754	1.17 (0.44–3.08)
carbapenems use	21 (70.0)	30 (76.9)	0.517	1.43 (0.49–4.20)
vancomycin use	4 (13.3)	6 (15.4)	0.811	1.18 (0.30–4.63)
multiple antibiotic use	19 (63.3)	18 (46.2)	0.158	0.50 (0.19–1.31)
antibiotic therapeutic duration (d)	28.1 (10.0, 46.2)	38.0 (19.3, 56.7)	0.033	
prophylaxis antifungal therapy	19 (63.3)	32 (82.1)	0.084	2.65 (0.88–7.99)
antifungal therapeutic duration (d)	7.9 (2.3, 13.5)	8.8 (2.3, 15.3)	0.535	
Outcome				
death	1 (3.3)	5 (12.8)	0.197	4.27 (0.47–38.62)

In the univariate logistic regression model, mechanical ventilation was significantly more common in neonates with candidemia caused by *non-C.albicans* (74.4% vs 46.7%, *P* = 0.021). The antibiotic therapeutic duration was longer in the *non-C.albicans* group compared to *C.albicans* group (38.0 vs 28.1 days, *P* = 0.033). Among infants with candidemia, 48.7% were conceived using assisted reproductive technology, compared with 16.7% in *C.albicans* group (*P* = 0.008).

Based on step-wise multivariate logistic regression analysis, infants with *non-C.albicans* candidemia infections were more likely to undergo mechanical ventilation (OR, 95%CI = 3.13, 1.07–9.14, *P* = 0.037). Infants who were conceived using assisted reproductive technology were more likely to develop *non-C.albicans* candidemia (OR, 95%CI = 4.52, 1.39–14.77, *P* = 0.012) (Table 2).

**Table 2:** Multivariate analysis for *C.albicans* and non-*C.albicans*

Risk factor	Odds ratio	95% CI	*P* value
assisted reproductive technology	4.52	1.39–14.77	0.012
mechanical ventilation	3.13	1.07–9.14	0.037

### Outcome

The overall mortality among affected infants was 5.8%. *C.albicans* and *non-C.albicans* were associated with a mortality rate of 3.3%, 12.8%, respectively. There was no significance between the two groups (*P* = 0.197).

## Discussion

*Candida* species have emerged as important pathogens which are associated with significant morbidity and mortality in neonates [1, 10]. In the past two decades, there have been several cohort studies indicating that the cause of *Candida* bloodstream infection has shifted from *C.albicans* to *non-C.albicans* [19–21]. Widespread use of azole antifungal agents, especially fluconazole, was one of the reasons contributing to the increased isolation rate of *non-C.albicans* and the decreased isolation rate of *C.albicans* [22, 23]. Many studies from western countries showed that *C.albicans* was the predominant *Candida* species isolated from candidemia, followed by *C.parapsilosis* among infants [3, 22]. It was reported that *C.parapsilosis* attributed to one-fifth of all cases of candidemia in neonates [24]. Our study revealed that *C.parapsilosis* attributed to only 1.4% of all cases of neonatal candidemia. In our study, we observed a changing pattern of neonatal candidemia among infant patients. The most frequent *Candida* species isolated from the bloodstream was *C.albicans* (43.5%). The most common *non-C.albicans* species was *C.glabrata* (33.3%), followed by *C.tropicalis* (20.3%). The *non-C.albicans* together accounted for more than 55% of all candidemia in our center. This species distribution pattern varied from western countries but was consistent with studies conducted in Australia and in 11 NICUs in China [21, 25]. It was reported that the difference of prevalence of candidemia due to a specific *Candida* specie may vary by geographic region [15], and some researches recommended that the study should focus at the local level rather than at the worldwide scale [26]. One of the outbreak of candidemia caused by *C.parapsilosis* reported from a Chinese hospital revealed that all strains was susceptible to fluconazole [27]. Fluconazole has been recommended as the best alternative antifungal drug for both of prophylaxis and primary treatment for patients with candidemia due to its efficacy and safety [28, 29]. In China, *C.parapsilosis* isolated from sterile body fluids have rarely been reported to be resistant to fluconazole [30, 31]. A prospective cohort study from a teaching hospital conducted between 2006 and 2011 reported that all 25 *C.parapsilosis* strains tested were susceptible to fluconazole [30]. The 2010 National China Hospital Invasive Fungal Surveillance Net (CHIF-NET) program found that only 1.4% of *C.parapsilosis* strains were resistant to fluconazole [31]. Based on those studies, we can state with certainty that *C.parapsilosis* specie isolated from blood stream is still highly susceptible to fluconazole in Chinese region and the use of fluconazole for prophylaxis in our hospital can be effective.

Many investigations have confirmed that use of broad-spectrum antibiotics and prolonged antibiotic therapeutic duration were the most common risk factors associated with neonatal candidemia [32]. A previous study showed that prolonged use of 3rd generation cephalosporin was associated with neonatal candidemia due to *C.parapsilosis* in infants [33]. Previous studies also suggested that prolonged antibiotic therapeutic duration predisposed to *C.glabrata* was attributed to candidemia [34, 35]. Our observation was in agreement with the general view that prolonged antibiotic use was associated with an increase of *non-C.albicians* candidemia in patients. It was reported that universal exposure to antibacterial drugs would suppress the bacterial flora and facilitate *Candida* colonization, which may increase the potential risk of development of candidemia [32]. These findings highlight the importance of evaluating the antibacterial burden in infected neonates.

In the multivariate logistic regression models, we found that mechanical ventilation affected the risk of candidemia infection with *non-C.albicans* isolates. It is well known that mechanical ventilation would play an important role in the pathogenesis of invasive *candidiasis* because of abrading the respiratory mucosa, providing *Candida* species for a portal of entry into the bloodstream [32]. Once *Candida* species adheres to the surface of invasive medical devices, it can form a biofilm thus protecting itself from immune responses and antifungal therapy.

Additionally, our analysis identified that use of assisted reproductive technology (ART) such as in vitro fertilization-assisted pregnancy would serve as an independent risk factor for development of *non-C.albicans* candidemia in newborns (OR, 95% CI = 4.52, 1.39–14.77, *P* = 0.012). This association may be partly due to the high proportion infection caused by *C.glabrata* among the neonates whom were the production of ART. In the *non-C.albicans* candidemia group, 73.7% (14/19) of neonates were conceived using ART. Globally, few studies have verified the association of ART and the outcome of neonatal candidemia. Most of the previous literature focused on the *Candida* species was associated with chorioamnionitis in in vitro fertilization (IVF) pregnancy [36]. It was reported that some types of uterine manipulation, such as IVF, may introduce the fungus at the time of embryo transfer [37]. Although *C.glabrata* showed a reduced virulence and absence of pathognomonic lesions compared with *C.albicans*, there was strong documented evidence of association between chorioamnionitis caused by *C.glabrata* and the use of ART, and the high lethality and morbidity in such group [38]. One of the studies showed that in 72% of cases of *C.glabrata* chorioamnionitis, conception was through ART [39]. These results raised the concern of the need for *Candida* species screening prior to IVF initialization. Although we failed to find out the data of culture results before embryo transfer, 73.9% of the infected neonates had received prophylaxis antifungal agents (fluconazole). Among the patients who were conceived using ART, 91.7% cases were administered fluconazole for prophylactic use. This may partly explain why the mortality rate was lower (overall mortality rate was 8.7%) that previous reports (24–30% in neonatal candidemia) [32].

The main limitation of our study was the retrospective design and the small sample size, which may compromise the statistical power. Additionally, we cannot collect the data of maternal yeasts colonization before and after delivery, which may not provide the enough information for evaluation of the vertical transmission and the association about maternal yeasts colonization and the subsequence of development of neonatal candidemia. Nevertheless, our data revealed that prolonged antibiotic therapeutic duration, mechanical ventilation and use of assisted reproductive technology were all the independent risk factors for the development of neonatal *non-C.albicans* candidemia.

## Conclusions

*Non-C.albicans* species are the major cause of candidemia in local neonatal group. The study highlights the urgent needs to evaluate the possibility of development of *non-C.albicans* candidemia in neonates exposed to these risk factors and much emphasis must be laid on the early implementation of medical intervention to reduce the incidences of candidemia in neonates.

## Abbreviations

95%CIs: 95% confidence intervals
ART: Assisted reproductive technology
*C.albicans*: *Candida albicans*
*C.glabrata*: *Candida glabrata*
*C.krusei*: *Candida krusei*
*C.parapsilosis*: *Candida parapsilosis*
*C.tropicalis*: *Candida tropicalis*
CHIF-NET: China Hospital Invasive Fungal Surveillance Net
IVF: in vitro fertilization
NICUs: Neonatal intensive care units
*non-C. albicans*: *non-Candida albicans*
ORs: Odds ratios
